# The Perivascular Fat Attenuation Index: Bridging Inflammation and Cardiovascular Disease Risk

**DOI:** 10.3390/jcm14134753

**Published:** 2025-07-04

**Authors:** Eliška Němečková, Kryštof Krása, Martin Malý

**Affiliations:** Department of Medicine, First Faculty of Medicine, Charles University in Prague and the Military University Hospital, 16902 Prague, Czech Republic; nemeckova.eliska@uvn.cz (E.N.); krystof.krasa@uvn.cz (K.K.)

**Keywords:** perivascular adipose tissue, pericoronary adipose tissue, fat attenuation index, coronary artery disease, inflammation, chronic inflammatory diseases, cardiometabolic risk, vascular inflammation

## Abstract

Cardiovascular disease remains the leading global cause of mortality, with inflammation now recognized as a central driver of atherosclerosis and other cardiometabolic conditions. Recent advances have repositioned perivascular adipose tissue from a passive structural element to an active endocrine and immunomodulatory organ, now a key focus in cardiovascular and metabolic research. Among the most promising tools for assessing perivascular adipose tissue inflammation is the fat attenuation index, a non-invasive imaging biomarker derived from coronary computed tomography angiography. This review explores the translational potential of the fat attenuation index for cardiovascular risk stratification and treatment monitoring in both coronary artery disease and systemic inflammatory or metabolic conditions (psoriasis, systemic lupus erythematosus, inflammatory bowel disease, obesity, type 2 diabetes, and non-obstructive coronary syndromes). We summarize evidence linking perivascular adipose tissue dysfunction to vascular inflammation and adverse cardiovascular outcomes. Clinical studies reviewing the fat attenuation index highlight its ability to detect subclinical inflammation and monitor treatment response. As research advances, standardization of measurement protocols and imaging thresholds will be essential for routine clinical implementation.

## 1. Introduction

Despite significant advances in cardiovascular imaging and preventive strategies, current risk prediction tools often fail to detect subclinical vascular inflammation, a key driver of atherosclerosis and cardiometabolic disease. This gap is particularly evident in patients with systemic inflammatory disorders or metabolic dysfunction, who remain under-recognized by conventional models. In recent years, FAI (fat attenuation index), a computed tomography-derived marker of perivascular inflammation, has emerged as a novel imaging biomarker with the potential to fill this gap [1].

Cardiovascular diseases (CVDs) remain the leading cause of mortality worldwide, accounting for an estimated 17.9 million deaths annually according to the World Health Organization. This burden is projected to rise to 35.6 million by 2050 [2], underscoring the urgent need for earlier and more precise risk-stratification tools. Studies have shown that FAI is relevant across a broad spectrum of chronic inflammatory and metabolic conditions. Chronic inflammatory diseases, such as inflammatory bowel disease, psoriasis, rheumatoid arthritis, and systemic lupus erythematosus, are associated with significantly elevated cardiovascular risk [3]. However, this risk is often inadequately captured by traditional scoring systems that rely on cholesterol levels, age, and blood pressure. This underscores the need for ongoing surveillance and early intervention, particularly in high-risk populations, before irreversible structural changes occur. Perivascular adipose tissue (PVAT), once considered a passive structural layer, is now viewed as an active participant in vascular health [4,5,6,7,8]. Systemic or local inflammation remodels PVAT, producing detectable changes in CT imaging quantified by FAI [6]. The interplay between PVAT and vascular inflammation, and how this interaction is captured via FAI on CT, is illustrated in the graphical abstract.

This review synthesizes the rapidly evolving literature on FAI, not only in the context of coronary artery disease but also in chronic inflammatory and cardiometabolic conditions. We explore its biological basis, methods of quantification, clinical applications, and the challenges that must be addressed to support its routine integration into cardiovascular care.

## 2. The Role of PVAT

**Figure 1:**
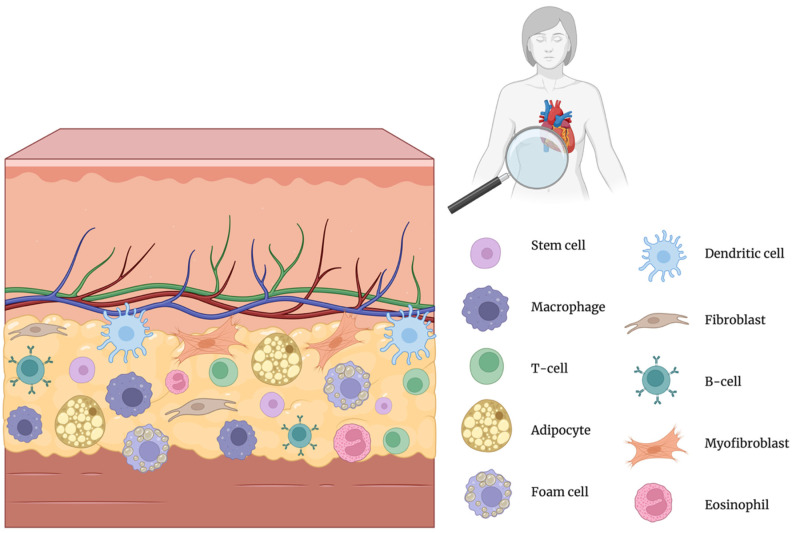
Cellular composition of perivascular adipose tissue (PVAT) [7]. Created in BioRender.com. Němečková, E. (2025). Available from: https://BioRender.com/ms2hh4k (accessed on 20 May 2025) [17].

Inflammation is a central driver of atherosclerosis, contributing to disease onset, progression, and cardiovascular complications [7]. Recent studies have highlighted the importance of PVAT as a novel biomarker for evaluating atherosclerosis and related vascular inflammation [8]. PVAT is a heterogeneous tissue composed not only of adipocytes but also of fibroblasts, mesenchymal stem cells, macrophages, eosinophils, and lymphocytes, as illustrated in Figure 1 [9]. Regional heterogeneity refers to significant variations in PVAT, depending on its anatomical location. PVAT surrounding distinct vascular beds, such as thoracic (tPVAT), abdominal (aPVAT), mesenteric (mPVAT), and coronary arteries (cPVAT or PCAT), differs substantially in adipocyte morphology, precursor origin, secretory profile, and inflammatory potential [10].

Morphologically, tPVAT contains multilocular brown adipocytes rich in mitochondria, while aPVAT and mPVAT consist predominantly of unilocular white adipocytes [11]. PCAT has a mixed “beige” phenotype with a high proportion of white adipocytes and features of brown fat, such as UCP1 expression [10,12,13]. Developmentally, different PVAT depots arise from distinct progenitor cells. For example, tPVAT develops partly from Myf5^+^ and SM22α^+^ progenitors, while aPVAT shares origins with vascular smooth muscle cells, and mPVAT may derive from visceral mesenchyme [10]. tPVAT produces fewer pro-inflammatory cytokines than aPVAT or mPVAT, which are more prone to diet-induced inflammation [5]. Functionally, tPVAT appears protective (anti-inflammatory, anti-atherogenic in mice), whereas aPVAT, mPVAT, and PCAT are associated with pro-inflammatory environments and atherosclerosis susceptibility [14].

Regional heterogeneity of PVAT fundamentally influences FAI measurement and interpretation. Biological, anatomical, and technical variability across coronary segments necessitates a standardized, region-specific approach to accurately interpret FAI as a biomarker of coronary inflammation and cardiovascular risk.

Given the regional differences in PVAT composition, baseline FAI values are expected to vary from one coronary segment to another—a reflection of local differences in adipocyte size, density, and water-to-lipid ratio. This biological heterogeneity is echoed in clinical outcomes: in CRISP-CT, FAI measured in the proximal RCA (right coronary artery) and LAD (left anterior descending artery) independently predicted both all-cause and cardiac mortality, whereas LCx (left circumflex artery)-derived values were less prognostic or pointed to different endpoints [15]. The RCA is often preferred for standardized FAI because its proximal segment has fewer anatomical variations, lacks large side branches, and is embedded in abundant fat within the coronary sulcus [16]. Vessel-specific reference ranges and AI-derived corrections (the FAI-Score) have been introduced to account for these anatomic and technical nuances. Beyond the coronary arteries, regional PVAT heterogeneity also explains why inflammatory signatures and CT-attenuation patterns differ between coronary and peripheral vessels [5].

Originally, it was thought that the development of atherosclerosis follows the “inside-out” model, which suggests that the process begins within the arterial wall due to endothelial dysfunction, inflammation, and foam cell formation. In this view, PVAT is considered a passive and supportive part of the vascular structure [4]. However, more recent research has revealed a complex relationship between PVAT and the blood vessel wall. The “outside-in” theory suggests that dysfunctional PVAT acts as a responsive sensor to inflammatory stimuli and communicates bi-directionally with the arterial wall [6]. Under normal circumstances, PVAT functions as an endocrine organ that promotes vascular homeostasis through vasodilatory, anti-oxidant, and anti-inflammatory effects [18]. Instead of the typically larger, well-differentiated adipocytes, systemic inflammation leads to the accumulation of smaller, less-differentiated adipocytes within the PVAT. A gradient forms, with the smallest adipocytes nearest to the inflamed vessel wall. This reflects a shift from a primary energy storage function to a more active secretory role [19].

Adiponectin, the most researched adipokine in PVAT, has diverse metabolic characteristics [20]. Adiponectin has potent anti-inflammatory and antioxidant properties and promotes vasodilation [21]. Interestingly, circulating vascular superoxide is connected to lower amounts of adiponectin and endothelial dysfunction. On the other hand, signs of oxidative stress cause adiponectin gene expression to increase in local PCAT. This is in contrast to other fat tissues, where oxidative stress degrades adiponectin, highlighting the unique physiology of PCAT [16]. By blocking the nuclear factor kappa B (NF-κB pathway), adiponectin helps to lower the expression of pro-inflammatory cytokines, including tumor necrosis factor alpha (TNFα) and interleukin-6 [22]. On the other hand, it stimulates the synthesis of anti-inflammatory cytokines such as IL-10 and IL-1RA [23]. The prominent adiponectin expression of PVAT [24] is undoubtedly linked to its anti-inflammatory influence on the surrounding vasculature. Inflammation leads to increased production of pro-inflammatory substances such as leptin, IL-6, TNF-α, IFN-γ, and monocyte chemoattractant protein-1 (MCP-1), which diffuse into the adjacent PVAT, further fueling the inflammatory response and ultimately leading to vascular dysfunction and an increased risk of developing CVD [1,25]. Beyond its outward signaling capacity, PVAT is also dynamically influenced by signals originating from the vascular wall, a process often referred to as “inside-out” signaling. For example, oxidative stress within the endothelium can trigger the release of lipid peroxidation products such as 4-hydroxynonenal (4-HNE), which diffuses into the adjacent PVAT and activates peroxisome proliferator-activated receptor gamma (PPARγ). This activation leads to enhanced secretion of adiponectin, which in turn exerts antioxidant and anti-inflammatory effects back on the vessel wall. This feedback loop highlights the integrated crosstalk between vascular and adipose tissues, where PVAT not only senses but also adapts to inflammatory and oxidative cues from its microenvironment [11,14,18,25].

PVAT dysfunction may also contribute to the development of hyperglycemia and type 2 diabetes [26]. This perspective highlights the importance of PVAT as an active player in the pathophysiology of atherosclerosis and a potential therapeutic target. Obesity profoundly alters the phenotype and function of PVAT, promoting a shift toward larger, lipid-rich white adipocytes and a pro-inflammatory secretory profile, driving vascular dysfunction from the outside in. Elevated FAI values in obese individuals have been consistently linked to increased vascular risk, even in the absence of overt coronary artery disease or calcified plaque. FAI, therefore, holds promise as a powerful tool for unmasking subclinical vascular inflammation. Early evidence suggests that FAI responds dynamically to metabolic interventions, including weight loss, improved glycemic control, and anti-inflammatory therapies. Applying FAI in the context of obesity demands careful interpretation. Differences in body habitus, PVAT distribution, and adipose tissue remodeling introduce biological variability that must be accounted for in clinical interpretation. Standardized approaches such as the FAI-Score may help overcome this barrier and pave the way for broader adoption of FAI in obesity-related cardiovascular risk prediction.

## 3. The Perivascular Fat Attenuation Index (pFAI)

**Figure 2:**
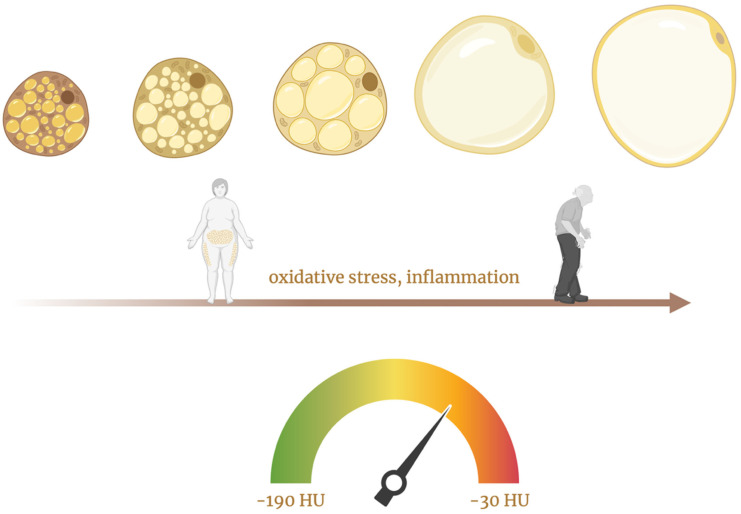
This figure illustrates the progressive remodeling of perivascular adipocytes in response to chronic stressors such as aging, obesity, and cardiometabolic disease, which drive oxidative stress and low-grade inflammation. These stimuli promote a transition from small, metabolically active, brown-like adipocytes to large, lipid-rich, white-like adipocytes with impaired function [5,26]. Created in BioRender.com. Němečková, E. (2025). Available from: https://BioRender.com/m0l9f3r (accessed on 20 May 2025) [27].

Extended exposure to inflammatory mediators impairs the normal differentiation of preadipocytes into mature adipocytes, affecting the intracellular accumulation of lipid droplets in these cells while also initiating lipolysis [14]. Vascular inflammation causes the composition of PVAT to change from a lipid-rich state to a more aqueous state. This shift leads to an increase in the density (attenuation) of PVAT on CT scans [6]. In a sense, PVAT functions as a “thermometer” for vascular inflammation [19]. This shift in tissue composition underlies the rationale for using FAI, a non-invasive CT-based biomarker that quantifies coronary inflammation by mapping attenuation gradients in pericoronary fat. This process is depicted in Figure 2, which illustrates the remodeling of perivascular adipocytes in response to chronic stressors such as aging, obesity, and cardiometabolic disease.

Antonopoulos et al. were the first to use and validate FAI via CT angiography [1]. This was in contrast to the typical evaluation of calcium content in vascular plaques via coronary calcium scoring. PVAT around the epicardial coronary arteries is commonly characterized as adipose tissue located within a radial distance from the outer wall of the vessel equal to the diameter of the neighboring coronary artery [1]. PCAT (pericoronary adipose tissue), the coronary equivalent of PVAT, includes all voxels ranging from −190 to −30 Hounsfield units (HU) inside a region of interest extending to an orthogonal distance equal to the diameter of the target vessel [12]. Some studies have quantified the FAI in defined proximal arterial segments, such as 40 mm from the origin of RCA, LAD, and LCx [28]. It is recommended to start FAI measurements on the RCA beyond the first 10 mm of its origin to minimize artifacts [28]. Initially, the FAI was assessed around the RCA’s proximal section, and later studies used algorithms to quantify the FAI around the proximal segments of the LAD and LCx [29]. The RCA typically has fewer collateral branches, more surrounding adipose tissue, and a relatively uniform lumen diameter from its origin to the distal segments, which facilitates standardized and reproducible measurement of FAI [12].

The typical HU values for PCAT range from −190 to −30 HU (relative to water attenuation at 0 HU) [12,29]. As mentioned, vascular inflammation can induce changes in adjacent PCAT, leading to lipolysis and a reduction in lipid accumulation. This shift results in a higher water-to-fat ratio, causing the HU values to become less negative (closer to −30 HU). Consequently, a higher FAI often indicates a greater level of coronary inflammation [6,30]. Table 1 summarizes the most frequently cited HU cut-offs in the recent literature. FAI measurements in HU are sensitive to multiple sources of variability. Altering tube voltage from 70 kV to 120 kV changes PCAT attenuation by ≈ 11 HU [31]; obesity yields hypertrophic, lipid-rich adipocytes with lower attenuation (more negative HU values), whereas lean subjects show higher baseline attenuation [5]. To overcome this heterogeneity, an AI-corrected FAI-Score has been introduced [19]. The algorithm automatically segments the coronary tree, measures three-dimensional attenuation gradients, and then adjusts for scanner voltage, age, sex, and artery-specific anatomy, outputting a unit-free score that is interpreted against age- and sex-stratified nomograms [29], enabling physicians to incorporate FAI into personalized prevention and therapeutic strategies. Such efforts will be critical for transitioning FAI from a research tool to a routine component of cardiovascular imaging reports.

The current methods are designed primarily for conventional CT scanners. FAI measurements on newer technologies such as photon-counting CT require further standardization and validation. A study by Mergen et al. indicated that epicardial adipose tissue attenuation and FAI measurements on photon-counting detector CT are affected by virtual monoenergetic image energy levels and the presence of a contrast agent [31]. Research has indicated that a VMI energy level of 70 keV results in fat attenuation values closest to those obtained with the conventional energy-integrating detector CT [31].

## 4. A New Frontier in Cardiovascular Care

The first study to investigate the prognostic potential of the pFAI was the CRISP-CT study. This post hoc analysis evaluated prospective data from two cohorts of approximately 4000 patients who were followed for up to ten years after CCTA (coronary computed tomography angiography) [15]. The pericoronary FAI values around the RCA and LAD proximal segment (but not around the LCx) were predictive of all-cause and cardiac mortality. The derivation cohort used −70.1 HU as the optimal FAI cut-off value. Values above this threshold were associated with an increase in mortality (9-fold for cardiac and 2.5-fold for all-cause), as well as a 5-fold increased risk of acute myocardial infarction. These findings were independent of sex, age, common risk factors, tube voltage, modified Duke CAD index, CCS (coronary artery calcium score), and HRP (high-risk plaque features). The predictive value of the FAI for cardiac mortality was lost in those who started taking statins and aspirin after CCTA, whereas among those who did not change their medication, the hazard ratio for cardiac mortality more than doubled. This finding shows that appropriate medical therapy may reduce the risk of FAI-detected CVD [15].

In continuation of this topic, the PARADIGM study, an international observational registry, investigated the progression of atherosclerotic plaques in relation to statin use. The participants included patients with suspected or confirmed coronary artery disease (CAD) who underwent serial CTA with a minimum interval of two years. Statins have been shown to change the composition of intraplaque components by increasing calcification and decreasing the amount of non-calcified plaque burden. This slows the progression of overall coronary atherosclerosis, with increased calcification in the plaque and fewer high-risk plaque features [36]. This finding is further supported by yet another study that reported a significant decrease in FAI scores after treatment with high-dose statins. These findings suggest that statins may exert an anti-inflammatory effect on PCAT [34].

The study by Suzuki et al. provides compelling evidence that inflammation significantly impacts the characteristics and potential instability of atherosclerotic plaques. Higher levels of non-calcified plaque are associated with PCAT inflammation, which can be detected through a decreased PCAT density [37]. Further studies also confirmed the ability of FAI to track unstable atherosclerotic plaques and culprit lesions in patients with acute coronary syndrome, which tend to exhibit higher FAI values than stable plaques do [29,37,38]. Additionally, hemodynamically significant plaques (defined as those with FFRs ≤ 0.8) are generally associated with high FAI values [39].

Another recent publication sought to answer the question of whether pFAI measured around the proximal RCA could predict future acute coronary events in patients with nonobstructive CAD. A single-center, prospective observational study included patients with atypical chest pain who underwent CCTA and were found to have nonobstructive CAD. A high RCA FAI of >−77.3 HU on CCTA was found to help identify high-risk patients who may need regular follow-up and early initiation of interventions [32]. These findings demonstrate the strong association between elevated FAI and adverse cardiovascular outcomes, ranging from unstable plaque characteristics in atherosclerotic disease to the prediction of acute coronary events in patients with non-obstructive CAD and even the identification of high-risk individuals presenting with Takotsubo syndrome and MINOCA [40]. The observed association strengthens the argument for its potential utility in identifying coronary inflammation in less typical contexts, as illustrated by its application to a case of HES-related INOCA (hypereosinophilic syndrome–related ischemia and non-obstructive coronary arteries). A case study successfully used FAI to detect coronary inflammation in a patient suffering from HES-related INOCA, a condition characterized by perfusion defects (on cardiac MRI) and vasospasm without obstructive CAD [41]. This observation suggests that the FAI might effectively identify inflammation as a primary driver of INOCA in patients with HES, highlighting its potential in diagnosing this condition.

Zuo et al. revealed that FAI is also sensitive to treatment-induced changes in coronary inflammation, detecting increases in non-target lesions despite an overall reduction in inflammation (which is credited to the anti-inflammatory medication given in connection with stenting). This suggests that stenting one coronary artery may lead to an increase in inflammation in other, untreated parts of the coronary vasculature [42]. This finding suggests that the FAI may be particularly useful in detecting clinically relevant, localized inflammation that could predict adverse events, such as in-stent restenosis [43]. FAI values could also potentially serve as a predictive biomarker for graft occlusion after CABG (coronary artery bypass graft). Huang et al. suggested that a higher FAI value in the right coronary artery before CABG surgery is associated with a greater risk of graft occlusion after surgery [44].

Notably, the relationship between FAI and traditional serum inflammatory markers remains unclear. Several studies, including those by Zhang et al. and Dai et al., have reported no significant correlation between FAI and markers such as hs-CRP or the absolute neutrophil count [35,45]. This lack of a strong correlation does not diminish the value of the FAI as a tool for measuring localized inflammation but rather underscores the importance of distinguishing between systemic and localized inflammation.

The success of IL-1β-targeted therapy (canakinumab) in reducing cardiovascular risk regardless of cholesterol levels [46] strongly suggests that FAI, with its ability to quantify localized coronary inflammation, may be a powerful tool for guiding treatment strategies and improving patient outcomes. Some antidiabetic therapies, such as GLP-1 receptor agonists, have been shown to promote adipocyte differentiation and reduce local inflammation within adipose tissue [26]. Additionally, statins, beyond their lipid-lowering properties, may exert direct anti-inflammatory effects on PVAT, as evidenced by reductions in FAI on follow-up CCTA [34]. These observations underscore the potential for developing PVAT-specific pharmacologic strategies, either through metabolic modulation or direct targeting of adipose inflammation.

## 5. Unravelling the Link to Systemic Inflammation

**Figure 3:**
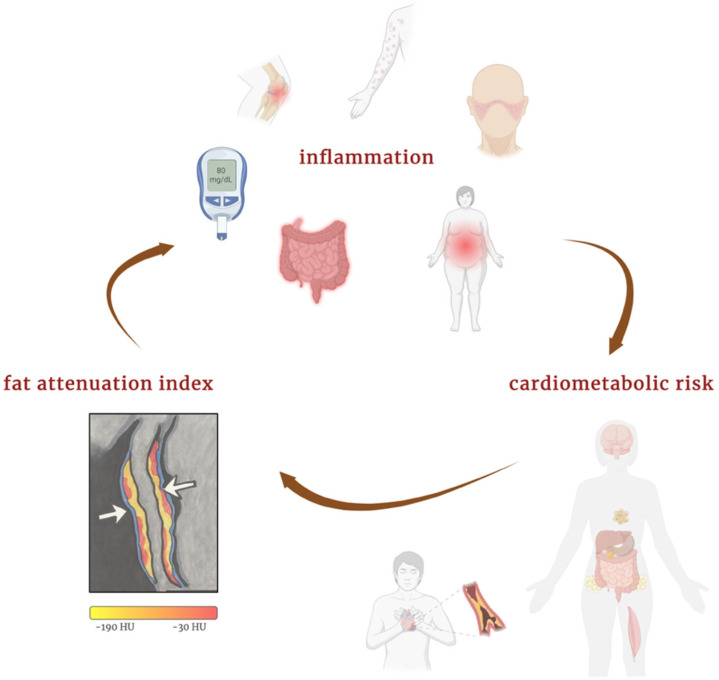
Interplay between inflammation, FAI, and cardiometabolic risk. Created in BioRender.com. Němečková, E. (2025) Available from: https://BioRender.com/qj5lw27 (accessed on 20 May 2025) [48].

Systemic inflammatory diseases are associated with an increased risk of cardiovascular events compared with the general population. This elevated risk includes cardiovascular mortality, non-fatal myocardial infarction, non-fatal stroke, and coronary revascularization [47]. In patients with immune-mediated inflammatory diseases, FAI could be a useful tool for identifying those at greater cardiovascular risk who might benefit from more intensive preventive strategies. Figure 3 illustrates the interplay between systemic inflammation, perivascular adipose tissue, and elevated FAI, highlighting how chronic immune activation contributes to cardiometabolic risk beyond traditional factors.

A comprehensive cardiovascular-risk assessment in systemic inflammatory diseases requires tools that capture systemic immune activation and focal coronary pathology. A comparative overview of currently available cardiovascular-risk stratification and treatment-monitoring tools in systemic inflammatory diseases (SIDs) is presented in Table 2. Conventional risk calculators such as SCORE2, Framingham Risk Score, or QRISK3 are inexpensive and quick to apply, yet they consistently under-estimate the excess risk conferred by chronic inflammation in rheumatoid arthritis, psoriasis, and related disorders [49,50,51,52]. Disease-activity indices such as the Psoriasis Area and Severity Index (PASI) or the Disease Activity Score in 28 joints (DAS-28) reliably quantify cutaneous or joint involvement. Yet they exhibit little or no predictive power for future cardiovascular events [50,53]. Anatomical imaging (CAC-scoring or standard CCTA) identifies established atherosclerosis but misses early, non-calcified inflammation. 18F-FDG-PET/CT targets metabolically active leukocytes and can visualize aortic inflammation, but its coronary application is hampered by myocardial tracer spill-over, high radiation dose, and price [54]. Circulating biomarkers (hs-CRP, IL-6, TNF-α) are inexpensive, repeatable measures of global inflammatory load. However, multiple prospective studies and meta-analyses show they correlate only modestly with future coronary events, exhibit high intra-individual variability, and display no or only weak correlation with FAI, illustrating their poor sensitivity for segment-level coronary inflammation [45,55]. Hence, while serum biomarkers remain useful for systemic disease surveillance, they are not sufficient for cardiovascular risk management.

By contrast, FAI derived from routine CCTA offers a segment-specific, quantitative read-out of coronary inflammation that complements plaque morphology and calcium scoring [1,55], predicts cardiac and all-cause mortality beyond traditional risk factors and high-risk plaque features [15], further improves risk reclassification when processed through AI-based FAI-Score algorithms [29], and declines promptly after effective biologic therapy, even when systemic markers such as hs-CRP or lipid profile remain unchanged [56], while requiring no additional scan time or radiotracer.

**Table 2:** Cardiovascular risk stratification and treatment-monitoring tools in SIDs.

Domain	Widely-Used Technique	Main Strengths	Key Limitations
Conventional risk scores	Framingham, SCORE2, QRISK3, etc.	Low cost, point-of-care use	Underestimate SID CV-risk by ≥30% [51,52,57]
Disease-activity indices	PASI (psoriasis), DAS-28 (RA), SLEDAI, CDAI (IBD)	Track primary-disease severity	Poor surrogate for CV-outcome
Systemic inflammatory biomarkers	hs-CRP, IL-6, TNF-α, etc.	Inexpensive, serial follow-up	Reflect global inflammation, poor correlation with coronary events or FAI values, high biological variability
Anatomical CT/US imaging	CAC-score, CCTA, carotid IMT	Quantifies plaque burden and morphology	Detects late, structural disease, insensitive to active inflammation, operator dependent
Molecular/functional imaging	18F-FDG-PET/CT, 18F-NaF-PET/CT	Direct signal of cellular activity	Expensive, limited availability, higher radiation, FDG spill-over in coronaries
Invasive plaque imaging	IVUS, OCT	Plaque anatomy	Invasive, focal, unsuitable for longitudinal screening
CT-derived FAI	FAI on routine CCTA	Segment-specific coronary-inflammation metric, therapy-responsive	Requires CCTA and AI-based standardization (FAI-Score)

Psoriasis, which affects approximately 125 million people worldwide, is characterized by chronic inflammation mediated mainly by T-helper cell types 1 and 17 [58,59]. The most common form is plaque psoriasis (psoriasis vulgaris), accounting for more than 80% of cases. The triad of IL-23-producing dendritic cells, IL-17-producing Th17 cells, and activated keratinocytes plays key roles [60]. The identified genes are involved in antigen presentation (HLA-Cw6), cytokine signaling (IL12B, IL23R), and interferon and NF-κB signaling [61]. Patients with psoriasis have a relatively high prevalence of traditional cardiovascular risk factors, and severe psoriasis increases the risk of cardiovascular death [62]. Studies suggest an independent association between psoriasis and myocardial infarction, ischemic heart disease, and stroke [62,63,64,65]. To date, measurements of FAI in patients with psoriasis have been conducted primarily through prospective cohort studies utilizing CCTA.

Studies in psoriasis patients have shown that biologic treatment can reduce coronary inflammation, as measured by FAI, even when traditional cardiometabolic risk factors such as lipid levels and HbA1c remain unchanged [66]. Elnabawi et al. studied patients diagnosed with moderate to severe psoriasis who underwent CCTA scans at baseline and after a one-year follow-up period. They reported that patients receiving biologic treatments (including anti-TNFα, anti-IL-12/23, or anti-IL-17) exhibited a significant FAI reduction after one year, suggesting decreased coronary inflammation. In contrast, patients in the control group who did not receive biologic treatment and were instead treated with topical medications or phototherapy showed no significant changes in FAI values during the same period [65]. These claims are supported by other studies. A pilot, randomized, double-blind, placebo-controlled phase 2a trial examined how orticumab, a fully human monoclonal antibody against oxidized LDL, affects coronary and skin inflammation in moderate to severe psoriasis patients at high cardiovascular risk. Orticumab treatment lowered the FAI score in all three major coronary arteries, mostly in the right coronary artery, compared with baseline (*p* = 0.01) and placebo (*p* = 0.02). The CaRi-Heart risk score indicated the potential for an almost 50% relative risk reduction in fatal cardiac events. However, orticumab did not significantly affect LDL, HDL, or triglyceride levels [67]. Therefore, FAI assessment could be valuable in evaluating the impact of biologic therapy on cardiovascular risk in psoriasis patients, even when traditional markers remain stable.

While studies have primarily focused on conditions such as psoriasis, the principles underlying the relevance of FAI to coronary inflammation also apply to other inflammatory diseases. Shi et al. also reported that the chronic low-grade systemic inflammation characteristic of autoimmune rheumatic disease (ARD) could extend to PVAT and induce vascular dysfunction, contributing to the increased risk of cardiovascular diseases in these patients [13]. Rheumatoid arthritis patients and CAD patients who underwent coronary artery bypass grafting had more chronic inflammatory infiltration and mononuclear cell infiltration in the aorta’s media and inner adventitia than did control patients [68]. The expression of pentraxin 3 (PTX3), a molecule produced at sites of inflammation, was observed in the endothelial cells and adipocytes of the perivascular adipose tissue (PVAT) in a study of patients with rheumatoid arthritis and CAD [69]. These findings suggest that inflamed adipocytes in PVAT may be a significant factor in the pathogenesis of CAD associated with RA patients. The currently available sources do not indicate that FAI is widely used in patients with rheumatoid arthritis, nor do they provide specific studies assessing its validity and clinical utility in this population. Patients with IBD (inflammatory bowel disease) have an increased risk of atherosclerotic cardiovascular disease (ASCVD) and are considered to have IBD as an independent risk factor for ASCVD [70]. Although there are no direct data on FAI in IBD patients, the strong connection between IBD and cardiovascular risk driven by chronic inflammation indicates that FAI could be a promising marker for future research in this area.

In contrast to IBD, where measurements of FAI are absent in the literature, there is some evidence of FAI in patients with systemic lupus erythematosus (SLE). Lupus patients have greater PVAT attenuation, indicating increased inflammation [71]. The FAI also predicts cardiovascular events in diabetic individuals. Compared with non-diabetic controls, diabetic patients have a greater pFAI [39], which independently predicts major adverse cardiovascular and cerebrovascular events [33]. A summary of studies evaluating FAI in systemic inflammatory diseases is provided in Table 3.

A higher FAI on CCTA is also associated with poorer cardiovascular outcomes in MASLD patients. Individuals with MASLD are recommended to undergo screening for liver fibrosis via the FIB-4 index in conjunction with HFS (hepamet fibrosis score) and LSM (liver stiffness measurement) [73]. FAI is also being investigated as a potential marker for assessing cardiovascular toxicity associated with chemotherapy. A post hoc analysis of a prospective study evaluated anthracycline-induced myocardial toxicity in breast cancer patients before they started anthracycline treatment and then at 3, 6, and 12 months after chemotherapy. The study revealed a significant, gradual increase in total FAI over 12 months compared with baseline [74].

## 6. Addressing the Limitations of FAI

While the FAI represents a non-invasive marker of coronary inflammation with the potential to improve cardiovascular risk prediction, several significant limitations must be mentioned.

One of the key obstacles is the technical complexity and limited availability of the necessary equipment. FAI analysis often requires specialized software and high-performance workstations, which may not be standard equipment in all clinical settings. This poses considerable logistical and financial challenges for the wide implementation of FAI in routine clinical practice. For the clinical use of the FAI, the establishment of clear reference and threshold values for treatment decision-making and prognosis is crucial. Current studies often use different cut-off points, and absolute FAI values can be influenced by technical factors, biological characteristics, and the anatomical location of measurement.

The potential widespread adoption of FAI would likely lead to an increase in the number of coronary CT angiographies performed or at least to the expansion of post-processing analysis of existing examinations. Although FAI itself does not increase the cost of examination if analyzed from an already performed CCTA, the argument for routinely performing CCTA in a broader population of patients with pro-inflammatory conditions would have to consider the cost of the examination, the risks associated with radiation exposure and contrast agent administration, and the proven clinical benefits of FAI.

Although observational data show consistent reductions in FAI after biologic agents (anti-TNF-α, anti-IL-12/23, anti-IL-17), high-dose statins, and the anti-oxLDL antibody orticumab, these findings should be viewed as hypothesis-generating [30,34,36,56,66,67]. Most studies were single-centre cohorts with limited sample size, short follow-up, and no adjudication of hard cardiovascular end-points, while randomized evidence is lacking. Importantly, no prospective trial has yet employed FAI either as an enrolment stratification tool or as a prespecified therapeutic end-point. Large, multicenter randomized clinical trials are required to determine whether modification of FAI translates into improved major adverse cardiovascular events and FAI-guided treatment allocation outperforms current algorithms based on traditional risk factors, calcium scoring, or plaque morphology.

## 7. Conclusions

Initially introduced to assess coronary inflammation, FAI is now recognized as a versatile biomarker applicable to a range of cardiometabolic and systemic inflammatory conditions. Its significance is also expanding to cancer therapy-related cardiotoxicity, diabetes, or autoimmune disorders, all associated with heightened cardiovascular risk. FAI detects localized vascular inflammation and captures treatment-induced changes, even when conventional risk markers remain unchanged. This sensitivity highlights its role in guiding personalized cardiovascular care, especially in patients whose risk is underestimated by traditional assessment models.

As interest in FAI grows, integrating the marker into routine clinical workflows represents a key objective for cardiovascular imaging research and practice. Tools such as the FAI-Score and AI-assisted quantification aim to standardize measurements across patient populations and imaging platforms. By detecting vascular inflammation early and tracking therapeutic effects, FAI may improve cardiovascular risk prediction and guide therapeutic decision-making. Embedding this metric into standard CCTA reports could immediately upgrade the management of “low-to-intermediate” risk patients by triggering earlier, better-targeted preventive therapy.

## Figures and Tables

**Table 1:** Commonly used HU thresholds for FAI.

Study	Patient Population/Scanner	Basic PCAT Window (HU)	“High-Risk” FAI Cut-Off
Antonopoulos et al. 2017 [1]	Mixed elective CCTA referrals, N = 273; 64-slice CT	–190 to –30	–70.1
CRISP-CT (Oikonomou 2018) [15]	Stable chest pain or screening, N = 3909; ≥64-slice CT	–190 to –30	–70.1
Sagris et al. 2022 (meta-analysis) [28]	Ten CCTA outcome studies, N ≈ 16,000 (pooled)	–190 to –30	–70.0
Biradar et al. 2025 [32]	Non-obstructive CAD with atypical chest pain, N = 302; 256-slice CT	–190 to –30	–77.3
Ding et al. 2025 [33]	Type-2 diabetes undergoing CCTA, N = 510; dual-source CT	–190 to –30	–75.0
Mátyás et al. 2024 [34]	Follow-up after ≥12 months high-dose statin, N = 118; 320-row CT	–190 to –30	–70.0
Zhang et al. 2024 [35]	New-onset chest pain, troponin-negative, N = 524; 128-slice CT	–190 to –30	–71.7

**Table 3:** Studies evaluating FAI in systemic inflammatory diseases.

Author (Year)	Study Type	Sample Size	Population	Main Finding	CT Modality
Elnabawi et al. 2019 [56]	Prospective cohort	82	Psoriasis patients	FAI significantly reduced after 1 year of biologic treatment	Conventional energy-integrating detector CT (EID-CT)
Farina et al. 2024 [67]	Randomized placebo-controlled trial	40	Psoriasis patients treated with orticumab	Orticumab reduced FAI despite unchanged lipid levels	Dual-source CT (Siemens Somatom Force)
Weber et al. 2023 [71]	Cross-sectional imaging study	54	Patients with systemic lupus erythematosus (SLE)	Higher perivascular HU values indicating inflammation	Third-generation dual-source CT (Somatom Force)
Karpouzas et al. 2021 [72]	Imaging sub-study (CIRT–Rheumatoid Arthritis)	130	Patients with rheumatoid arthritis	Higher FAI values correlated with systemic inflammation and disease activity	Sixty-four-slice CT scanner (GE Discovery)

## Data Availability

No new data were generated or analyzed in this narrative review. All information discussed is derived from previously published, publicly available sources cited in the reference list. Accordingly, a data-sharing statement is not applicable.

## Abbreviations

The following abbreviations are used in this manuscript:

AI	Artificial Intelligence
ARD	Autoimmune Rheumatic Disease
ASCVD	Atherosclerotic Cardiovascular Disease
CABG	Coronary Artery Bypass Graft
CAD	Coronary Artery Disease
CAC	Coronary artery calcium
CCS	Coronary artery Calcium Score
CCTA	Coronary Computed Tomography Angiography
CDAI	Crohn’s Disease Activity Index
CRISP-CT	Cardiovascular RISk Prediction using Computed Tomography
CT	Computed Tomography
CVD	Cardiovascular Disease
DAS-28	Disease Activity Score in 28 joints
FAI	Fat Attenuation Index
FFR	Fractional Flow Reserve
FIB-4	Fibrosis-4 Index for Liver Fibrosis
18F-FDG-PET/CT	Positron-emission tomography/CT with 18F-fluorodeoxyglucose
HES	Hypereosinophilic Syndrome
HFS	Hepamet Fibrosis Score
HLA	Human Leukocyte Antigen
HRP	High-Risk Plaque
HU	Hounsfield Units
IBD	Inflammatory Bowel Disease
IFN-γ	Interferon-gamma
IL-1RA	Interleukin-1 Receptor Antagonist
IL-6	Interleukin 6
IL-10	Interleukin 10
IL-12/23	Interleukin 12 and 23
IL-17	Interleukin 17
IL-1β	Interleukin-1 Beta
IL-23	Interleukin 23
IMT	Intima–media thickness
IVUS	Intravascular ultrasound
INOCA	Ischemia and Non-Obstructive Coronary Arteries
LAD	Left Anterior Descending Artery
LCx	Left Circumflex Artery
LDL	Low-Density Lipoprotein
LSM	Liver Stiffness Measurement
MASLD	Metabolic dysfunction-associated steatotic liver disease
MCP-1	Monocyte Chemoattractant Protein-1
MINOCA	Myocardial Infarction with Non-Obstructive Coronary Arteries
8F-NaF-PET/CT	PET/CT with 18F-sodium fluoride
NF-κB	Nuclear Factor Kappa B
OCT	Optical coherence tomography
PASI	Psoriasis Area and Severity Index
PET/CT	Positron-emission tomography/computed tomography
pFAI	Perivascular Fat Attenuation Index
PCAT	Pericoronary Adipose Tissue
PTX3	Pentraxin 3
PVAT	Perivascular Adipose Tissue
tPVAT	Thoracic Periaortic Adipose Tissue
aPVAT	Abdominal Periaortic Adipose Tissue
mPVAT	Mesenteric Perivascular Adipose Tissue
QRISK3	QRESEARCH risk estimator version 3
RA	Rheumatoid Arthritis
RCA	Right Coronary Artery
SCORE2	European Systematic Coronary Risk Evaluation, version 2
SIDs	Systemic inflammatory diseases
SLE	Systemic Lupus Erythematosus
SLEDAI	Systemic Lupus Erythematosus Disease Activity Index
TNF-α	Tumor Necrosis Factor Alpha
VMI	Virtual Monoenergetic Image

## References

[1] Antonopoulos A.S., Sanna F., Sabharwal N., Thomas S., Oikonomou E.K., Herdman L., Margaritis M., Shirodaria C., Kampoli A.-M., Akoumianakis I. (2017). Detecting human coronary inflammation by imaging perivascular fat. Sci. Transl. Med..

[2] Chong B., Jayabaskaran J., Jauhari S.M., Chan S.P., Goh R., Kueh M.T.W., Li H., Chin Y.H., Kong G., Anand V.V. (2024). Global burden of cardiovascular diseases: Projections from 2025 to 2050. Eur. J. Prev. Cardiol..

[3] Fernández-Gutiérrez B., Perrotti P.P., Gisbert J.P., Domènech E., Fernández-Nebro A., Cañete J.D., Ferrándiz C., Tornero J., García-Sánchez V., Panés J. (2017). Cardiovascular disease in immune-mediated inflammatory diseases. Medicine.

[4] Nava E., Llorens S. (2019). The Local Regulation of Vascular Function: From an Inside-Outside to an Outside-Inside Model. Front. Physiol..

[5] Li X., Ma Z., Zhu Y.Z. (2021). Regional Heterogeneity of Perivascular Adipose Tissue: Morphology, Origin, and Secretome. Front. Pharmacol..

[6] Simantiris S., Pappa A., Papastamos C., Korkonikitas P., Antoniades C., Tsioufis C., Tousoulis D. (2024). Perivascular Fat: A Novel Risk Factor for Coronary Artery Disease. Diagnostics.

[7] Ajoolabady A., Pratico D., Lin L., Mantzoros C.S., Bahijri S., Tuomilehto J., Ren J. (2024). Inflammation in atherosclerosis: Pathophysiology and mechanisms. Cell Death Dis..

[8] Antoniades C., Tousoulis D., Vavlukis M., Fleming I., Duncker D.J., Eringa E., Manfrini O., Antonopoulos A.S., Oikonomou E., Padró T. (2023). Perivascular adipose tissue as a source of therapeutic targets and clinical biomarkers. Eur. Heart J..

[9] Sigdel S., Udoh G., Albalawy R., Wang J. (2024). Perivascular Adipose Tissue and Perivascular Adipose Tissue-Derived Extracellular Vesicles: New Insights in Vascular Disease. Cells.

[10] Grigoras A., Amalinei C., Balan R.A., Giusca S.E., Caruntu I.D. (2019). Perivascular adipose tissue in cardiovascular diseases—An update. Anatol. J. Cardiol..

[11] Li Y., Chen Z., Xiao Y., Li X. (2024). Cross-talks between perivascular adipose tissue and neighbors: Multifaceted nature of nereids. Front. Pharmacol..

[12] Yuvaraj J., Cheng K., Lin A., Psaltis P.J., Nicholls S.J., Wong D.T.L. (2021). The Emerging Role of CT-Based Imaging in Adipose Tissue and Coronary Inflammation. Cells.

[13] Shi H., Wu H., Winkler M.A., de Chantemèle E.J.B., Lee R., Kim H.W., Weintraub N.L. (2022). Perivascular adipose tissue in autoimmune rheumatic diseases. Pharmacol. Res..

[14] Muffová B., Králová Lesná I., Poledne R. (2024). Physiology and Pathobiology of Perivascular Adipose Tissue: Inflammation-Based. Physiol. Res..

[15] Oikonomou E.K., Marwan M., Desai M.Y., Mancio J., Alashi A., Hutt Centeno E., Thomas S., Herdman L., Kotanidis C.P., Thomas K.E. (2018). Non-invasive detection of coronary inflammation using computed tomography and prediction of residual cardiovascular risk (the CRISP CT study): A post-hoc analysis of prospective outcome data. Lancet Lond. Engl..

[16] Tan N., Dey D., Marwick T.H., Nerlekar N. (2023). Pericoronary Adipose Tissue as a Marker of Cardiovascular Risk: JACC Review Topic of the Week. J. Am. Coll. Cardiol..

[17] Němečková E. (2025). Figure 1. [Internet]. https://BioRender.com/ms2hh4k.

[18] Rami A.Z.A., Hamid A.A., Anuar N.N.M., Aminuddin A., Ugusman A. (2022). Exploring the Relationship of Perivascular Adipose Tissue Inflammation and the Development of Vascular Pathologies. Mediat. Inflamm..

[19] Antoniades C., Kotanidis C.P., Berman D.S. (2019). State-of-the-art review article. Atherosclerosis affecting fat: What can we learn by imaging perivascular adipose tissue?. J. Cardiovasc. Comput. Tomogr..

[20] Sowka A., Dobrzyn P. (2021). Role of Perivascular Adipose Tissue-Derived Adiponectin in Vascular Homeostasis. Cells.

[21] Lei X., Qiu S., Yang G., Wu Q. (2023). Adiponectin and metabolic cardiovascular diseases: Therapeutic opportunities and challenges. Genes Dis..

[22] Ajuwon K.M., Spurlock M.E. (2005). Adiponectin inhibits LPS-induced NF-kappaB activation and IL-6 production and increases PPARgamma2 expression in adipocytes. Am. J. Physiol. Regul. Integr. Comp. Physiol..

[23] Wolf A.M., Wolf D., Rumpold H., Enrich B., Tilg H. (2004). Adiponectin induces the anti-inflammatory cytokines IL-10 and IL-1RA in human leukocytes. Biochem. Biophys. Res. Commun..

[24] Gruzdeva O., Dyleva Y., Belik E., Sinitsky M., Stasev A., Kokov A., Brel N., Krivkina E., Bychkova E., Tarasov R. (2022). Relationship between Epicardial and Coronary Adipose Tissue and the Expression of Adiponectin, Leptin, and Interleukin 6 in Patients with Coronary Artery Disease. J. Pers. Med..

[25] Nosalski R., Guzik T.J. (2017). Perivascular adipose tissue inflammation in vascular disease. Br. J. Pharmacol..

[26] Queiroz M., Sena C.M. (2024). Perivascular adipose tissue: A central player in the triad of diabetes, obesity, and cardiovascular health. Cardiovasc. Diabetol..

[27] Němečková E. (2025). Figure 2. [Internet]. https://BioRender.com/m0l9f3r.

[28] Sagris M., Antonopoulos A.S., Simantiris S., Oikonomou E., Siasos G., Tsioufis K., Tousoulis D. (2022). Pericoronary fat attenuation index—A new imaging biomarker and its diagnostic and prognostic utility: A systematic review and meta-analysis. Eur. Heart J.-Cardiovasc. Imaging.

[29] Oikonomou E.K., Antonopoulos A.S., Schottlander D., Marwan M., Mathers C., Tomlins P., Siddique M., Klüner L.V., Shirodaria C., Mavrogiannis M.C. (2021). Standardized measurement of coronary inflammation using cardiovascular computed tomography: Integration in clinical care as a prognostic medical device. Cardiovasc. Res..

[30] Van Der Bijl P., Kuneman J.H., Bax J.J. (2022). Pericoronary adipose tissue attenuation: Diagnostic and prognostic implications. Eur. Heart J.-Cardiovasc. Imaging.

[31] Mergen V., Ried E., Allmendinger T., Sartoretti T., Higashigaito K., Manka R., Euler A., Alkadhi H., Eberhard M. (2021). Epicardial Adipose Tissue Attenuation and Fat Attenuation Index: Phantom Study and In Vivo Measurements With Photon-Counting Detector CT. Am. J. Roentgenol..

[32] Biradar B., Valakkada J., Ayappan A., Kannath S., Sasidharan B., Alex A. (2025). Right coronary artery pericoronary fat attenuation index as a future predictor for acute coronary events in nonobstructive coronary artery disease—A prospective single centre study. Clin. Radiol..

[33] Ding Y., Shan D., Han T., Liu Z., Wang X., Dou G., Xin R., Guo Z., Chen G., Jing J. (2025). Incremental Prognostic Value of Perivascular Fat Attenuation Index in Patients with Diabetes with Coronary Artery Disease. Radiol. Cardiothorac. Imaging.

[34] Mátyás B.B., Benedek I., Raț N., Blîndu E., Parajkó Z., Mihăilă T., Benedek T. (2024). Assessing the Impact of Long-Term High-Dose Statin Treatment on Pericoronary Inflammation and Plaque Distribution—A Comprehensive Coronary CTA Follow-Up Study. Int. J. Mol. Sci..

[35] Zhang X., Cao Z., Xu J., Guan X., He H., Duan L., Ji L., Liu G., Guo Q., You Y. (2024). Peri-coronary fat attenuation index combined with high-risk plaque characteristics quantified from coronary computed tomography angiography for risk stratification in new-onset chest pain individuals without acute myocardial infarction. PLoS ONE.

[36] Lee S.-E., Chang H.-J., Sung J.M., Park H.-B., Heo R., Rizvi A., Lin F.Y., Kumar A., Hadamitzky M., Kim Y.J. (2018). Effects of Statins on Coronary Atherosclerotic Plaques: The PARADIGM Study. JACC Cardiovasc. Imaging.

[37] Suzuki K., Kinoshita D., Yuki H., Niida T., Sugiyama T., Yonetsu T., Araki M., Nakajima A., Seegers L.M., Dey D. (2024). Higher Noncalcified Plaque Volume Is Associated with Increased Plaque Vulnerability and Vascular Inflammation. Circ. Cardiovasc. Imaging.

[38] Kuneman J.H., van Rosendael S.E., van der Bijl P., van Rosendael A.R., Kitslaar P.H., Reiber J.H.C., Jukema J.W., Leon M.B., Ajmone Marsan N., Knuuti J. (2023). Pericoronary Adipose Tissue Attenuation in Patients with Acute Coronary Syndrome Versus Stable Coronary Artery Disease. Circ. Cardiovasc. Imaging.

[39] Yu Y., Ding X., Yu L., Dai X., Wang Y., Zhang J. (2022). Increased coronary pericoronary adipose tissue attenuation in diabetic patients compared to non-diabetic controls: A propensity score matching analysis. J. Cardiovasc. Comput. Tomogr..

[40] Gaibazzi N., Martini C., Botti A., Pinazzi A., Bottazzi B., Palumbo A.A. (2019). Coronary Inflammation by Computed Tomography Pericoronary Fat Attenuation in MINOCA and Tako-Tsubo Syndrome. J. Am. Heart Assoc..

[41] Port J.J., Weber B.N., Kadiyala M. (2025). Inflammation and INOCA: Can Fat Attenuation Indexing by Coronary CT Angiography Help Identify Coronary Inflammation?. JACC Case Rep..

[42] Zuo L., Tian Z., Zhou B., Hou M., Chen Y., Han P., Ma C., Wu X., Yu D. (2024). Perivascular fat attenuation index value and plaque volume increased in non-target lesions of coronary arteries after stenting. Eur. Radiol..

[43] Adolf R., Krinke I., Datz J., Cassese S., Kastrati A., Joner M., Schunkert H., Wall W., Hadamitzky M., Engel L.-C. (2025). Specific calcium deposition on pre-procedural CCTA at the time of percutaneous coronary intervention predicts in-stent restenosis in symptomatic patients. J. Cardiovasc. Comput. Tomogr..

[44] Huang S., Yu X., Yang B., Xu T., Gu H., Wang X. (2024). Predictive value of pericoronary fat attenuation index for graft occlusion after coronary artery bypass grafting. Jpn. J. Radiol..

[45] Dai X., Deng J., Yu M., Lu Z., Shen C., Zhang J. (2020). Perivascular fat attenuation index and high-risk plaque features evaluated by coronary CT angiography: Relationship with serum inflammatory marker level. Int. J. Cardiovasc. Imaging.

[46] Ridker P.M., Everett B.M., Thuren T., MacFadyen J.G., Chang W.H., Ballantyne C., Fonseca F., Nicolau J., Koenig W., Anker S.D. (2017). Antiinflammatory Therapy with Canakinumab for Atherosclerotic Disease. N. Engl. J. Med..

[47] Asenjo-Lobos C., González L., Bulnes J.F., Roque M., Muñoz Venturelli P., Rodríguez G.M. (2024). Cardiovascular events risk in patients with systemic autoimmune diseases: A prognostic systematic review and meta-analysis. Clin. Res. Cardiol..

[48] Němečková E. (2025). Figure 3. [Internet]. https://BioRender.com/qj5lw27.

[49] Crowson C.S., Matteson E.L., Roger V.L., Therneau T.M., Gabriel S.E. (2012). Usefulness of Risk Scores to Estimate the Risk of Cardiovascular Disease in Patients with Rheumatoid Arthritis. Am. J. Cardiol..

[50] Crowson C.S., Gabriel S.E., Semb A.G., Van Riel P.L.C.M., Karpouzas G., Dessein P.H., Hitchon C., Pascual-Ramos V., Kitas G.D., Trans-Atlantic Cardiovascular Consortium for Rheumatoid Arthritis (2017). Rheumatoid arthritis-specific cardiovascular risk scores are not superior to general risk scores: A validation analysis of patients from seven countries. Rheumatology.

[51] Zhu L., Singh M., Lele S., Sahakian L., Grossman J., Hahn B., McMahon M. (2022). Assessing the validity of QRISK3 in predicting cardiovascular events in systemic lupus erythematosus. Lupus Sci. Med..

[52] Colaco K., Ocampo V., Ayala A.P., Harvey P., Gladman D.D., Piguet V., Eder L. (2020). Predictive Utility of Cardiovascular Risk Prediction Algorithms in Inflammatory Rheumatic Diseases: A Systematic Review. J. Rheumatol..

[53] Innala L., Möller B., Ljung L., Magnusson S., Smedby T., Södergren A., Öhman M.-L., Rantapää-Dahlqvist S., Wållberg-Jonsson S. (2011). Cardiovascular events in early RA are a result of inflammatory burden and traditional risk factors: A five year prospective study. Arthritis Res. Ther..

[54] Tarkin J.M., Joshi F.R., Evans N.R., Chowdhury M.M., Figg N.L., Shah A.V., Starks L.T., Martin-Garrido A., Manavaki R., Yu E. (2017). Detection of Atherosclerotic Inflammation by ^68^Ga-DOTATATE PET Compared to [^18^F]FDG PET Imaging. J. Am. Coll. Cardiol..

[55] Antoniades C., Antonopoulos A.S., Deanfield J. (2020). Imaging residual inflammatory cardiovascular risk. Eur. Heart J..

[56] Elnabawi Y.A., Oikonomou E.K., Dey A.K., Mancio J., Rodante J.A., Aksentijevich M., Choi H., Keel A., Erb-Alvarez J., Teague H.L. (2019). Association of Biologic Therapy With Coronary Inflammation in Patients With Psoriasis as Assessed by Perivascular Fat Attenuation Index. JAMA Cardiol..

[57] Weber B., Liao K.P., DiCarli M., Blankstein R. (2021). Cardiovascular Disease Prevention in Individuals with Underlying Chronic Inflammatory Disease. Curr. Opin. Cardiol..

[58] Dairov A., Issabekova A., Sekenova A., Shakhatbayev M., Ogay V. (2024). Prevalence, incidence, gender and age distribution, and economic burden of psoriasis worldwide and in Kazakhstan. J. Clin. Med. Kazakhstan.

[59] Nussbaum L., Chen Y.L., Ogg G.S. (2021). Role of regulatory T cells in psoriasis pathogenesis and treatment. Br. J. Dermatol..

[60] Fitch E., Harper E., Skorcheva I., Kurtz S.E., Blauvelt A. (2007). Pathophysiology of Psoriasis: Recent Advances on IL-23 and Th17 Cytokines. Curr. Rheumatol. Rep..

[61] Sieminska I., Pieniawska M., Grzywa T.M. (2024). The Immunology of Psoriasis—Current Concepts in Pathogenesis. Clin. Rev. Allergy Immunol..

[62] Liu L., Cui S., Liu M., Huo X., Zhang G., Wang N. (2022). Psoriasis Increased the Risk of Adverse Cardiovascular Outcomes: A New Systematic Review and Meta-Analysis of Cohort Study. Front. Cardiovasc. Med..

[63] Ahlehoff O., Gislason G.H., Charlot M., Jørgensen C.H., Lindhardsen J., Olesen J.B., Abildstrøm S.Z., Skov L., Torp-Pedersen C., Hansen P.R. (2011). Psoriasis is associated with clinically significant cardiovascular risk: A Danish nationwide cohort study. J. Intern. Med..

[64] Gelfand J.M., Neimann A.L., Shin D.B., Wang X., Margolis D.J., Troxel A.B. (2006). Risk of myocardial infarction in patients with psoriasis. JAMA.

[65] Prodanovich S., Kirsner R.S., Kravetz J.D., Ma F., Martinez L., Federman D.G. (2009). Association of psoriasis with coronary artery, cerebrovascular, and peripheral vascular diseases and mortality. Arch. Dermatol..

[66] Smith A., Karahasan A., Yi D., Yapabandara S., Elhindi J., Fernandez-Penas P., Chow C., Zaman S. (2025). Biologic Therapy and Cardiometabolic Risk in Psoriasis: A Retrospective Review. Dermatol. Ther..

[67] Farina C.J., Davidson M.H., Shah P.K., Stark C., Lu W., Shirodaria C., Wright T., Antoniades C.A., Nilsson J., Mehta N.N. (2024). Inhibition of oxidized low-density lipoprotein with orticumab inhibits coronary inflammation and reduces residual inflammatory risk in psoriasis: A pilot randomized, double-blind placebo-controlled trial. Cardiovasc. Res..

[68] Hollan I., Scott H., Saatvedt K., Prayson R., Mikkelsen K., Nossent H.C., Kvelstad I.L., Liang M.H., Førre O.T. (2007). Inflammatory rheumatic disease and smoking are predictors of aortic inflammation: A controlled study of biopsy specimens obtained at coronary artery surgery. Arthritis Rheum..

[69] Hollan I., Nebuloni M., Bottazzi B., Mikkelsen K., Førre O.T., Almdahl S.M., Mantovani A., Fagerland M.W., Aukrust P., Meroni P.L. (2013). Pentraxin 3, a novel cardiovascular biomarker, is expressed in aortic specimens of patients with coronary artery disease with and without rheumatoid arthritis. Cardiovasc. Pathol. Off. J. Soc. Cardiovasc. Pathol..

[70] Cainzos-Achirica M., Glassner K., Zawahir H.S., Dey A.K., Agrawal T., Quigley E.M.M., Abraham B.P., Acquah I., Yahya T., Mehta N.N. (2020). Inflammatory Bowel Disease and Atherosclerotic Cardiovascular Disease: JACC Review Topic of the Week. J. Am. Coll. Cardiol..

[71] Weber B.N., Paik J.J., Aghayev A., Klein A.L., Mavrogeni S.I., Yu P.B., Mukherjee M. (2023). Novel Imaging Approaches to Cardiac Manifestations of Systemic Inflammatory Diseases: JACC Scientific Statement. J. Am. Coll. Cardiol..

[72] Karpouzas G.A., Rezaeian P., Ormseth S.R., Hollan I., Budoff M.J. (2021). Epicardial Adipose Tissue Volume As a Marker of Subclinical Coronary Atherosclerosis in Rheumatoid Arthritis. Arthritis Rheumatol..

[73] Zheng H., Sechi L.A., Navarese E.P., Casu G., Vidili G. (2024). Metabolic dysfunction-associated steatotic liver disease and cardiovascular risk: A comprehensive review. Cardiovasc. Diabetol..

[74] Kidoh M., Oda S., Sueta D., Egashira K., Hayashi H., Nakaura T., Nagayama Y., Yamamoto Y., Tsujita K., Hirai T. (2025). Serial assessment of coronary artery inflammation using cardiac CT in anthracycline chemotherapy for breast cancer. Eur. Radiol..

